# Meta-analysis of arterial spin labeling MRI to identify residual cerebral arteriovenous malformations after treatment

**DOI:** 10.1186/s12880-025-01668-3

**Published:** 2025-04-18

**Authors:** Shurun Wan, Xiuyan Yang, Yudi Zhuo, Fei Chen, Peiyue He, Weibo Luo, Yi Shi, Lingqun Zhu

**Affiliations:** 1 Key Laboratory of Chinese Internal Medicine of Educational Ministry and Beijing, Dongzhimen Hospital, Beijing University of Chinese Medicine, Beijing, 100700 China; 2 School of Chinese Medicine, Beijing University of Chinese Medicine, Beijing, 100029 China; 3 Dongzhimen Hospital, Beijing University of Chinese Medicine, Beijing, 100700 China

**Keywords:** Arterial spin labeling, Brain arteriovenous malformations, Meta-analysis

## Abstract

**Background:**

To use of statistical methods to assess the diagnostic value of arterial spin labeling (ASL) imaging for follow-up of treated arteriovenous malformations.

**Methods:**

We screened references from four databases, namely, the Cochrane Library, PubMed, Web of Science and Embase, that met the requirements. The methodology quality of the included studies was evaluated using the QUADAS-2 (Quality Assessment of Diagnostic Accuracy Studies-2) tool. Data pertaining to diagnostic performance were extracted, and the pooled sensitivity and specificity were calculated using a bivariate mixed-effects model.

**Results:**

We included six studies with a total of 132 patients with arteriovenous malformation (AVM). The merged sensitivity and specificity of ASL for the diagnosis of brain AVMs with incomplete occlusion after treatment were 0.94[0.86–0.98] and 0.99 [0.59-1.00], respectively. According to the SROC curve summary, the AUC was found to be 0.98 [0.96–0.99]. No significant publication bias was observed.

**Conclusion:**

While ASL does not currently match the diagnostic precision of DSA, it is instrumental in post-treatment surveillance of AVM patients. With the development of ASL technology in the future, this technique holds promise as a minimally invasive diagnostic strategy for AVMs with fewer side effects.

**Registration number of PROSPERO:**

CRD42023422087.

**Clinical trial number:**

Not applicable.

**Supplementary Information:**

The online version contains supplementary material available at 10.1186/s12880-025-01668-3.

## Background

Arteriovenous malformation (AVM) is a pathological condition arising from congenital vascular developmental anomalies, and is characterized by the absence of a capillary network or the presence of immature vascular networks between arteries and veins [1]. This disease can induce various clinical manifestations, including chronic headaches, migraines, and epileptic seizures [2, 3]. In severe cases, it may cause intracranial hemorrhage, leading to disability or even death [4, 5]. Despite the availability of various treatment modalities for AVM, such as surgical resection, radiation therapy, and endovascular treatment (EVT), there remains a significant issue of high recurrence rates among AVM patients’ post-treatment. Approximately 25% of patients who undergo EVT experience AVM recurrence within the first year following treatment [6], indicating that achieving the anticipated therapeutic outcomes is often a prolonged and challenging process [7]. Therefore, regular and precise radiological follow-ups are crucial for patients undergoing AVM treatment to assess the closure status of the lesion and monitor for recurrence.

Digital subtraction angiography (DSA) has long been considered the gold standard for diagnosing AVM due to its high-resolution and high-contrast vascular imaging capabilities [8, 9]. However, DSA is an invasive diagnostic method with significant limitations, including exposure to ionizing radiation, potential allergic reactions to iodinated contrast agents, and other complications [10, 11], as well as the discomfort and invasive nature of the procedure. These factors may adversely affect the patient, making it unsuitable for long-term follow-up examinations. Given the potential risks associated with DSA, arterial spin labeling (ASL) technology has emerged as a novel noninvasive vascular imaging method extensively used in the field of neuroimaging. ASL technology manipulates the spin state of protons in arterial blood, using blood as an endogenous tracer in conjunction with specific magnetic resonance imaging (MRI) sequences for vascular imaging [12, 13]. This clinical technique does not require the use of exogenous contrast agents and does not involve ionizing radiation, allowing patients to safely undergo repeated examinations. This is particularly important for long-term follow-up of patients post-AVM treatment. This study aimed to determine the accuracy and clinical utility of ASL in diagnosing the closure status of treated AVMs and in monitoring for recurrence.

## Method

Prior to initiating this study, it was already registered on PROSPERO under number CRD42023422087. This report refers to the Preferred Reporting Items for Systematic Reviews and Meta-Analyses of Diagnostic Test Accuracy Studies (PRISMA-DTA) guidelines [14, 15].

### Retrieval strategy

Four databases, the Cochrane Library, PubMed, Web of Science and Embase, were searched comprehensively from the inception of the database to the present. Our search strategy was not limited by publication date or language to ensure a comprehensive and inclusive literature retrieval. The search strategy employed “Arterial Spin Labeling” and “Intracranial Arteriovenous Malformations” as Medical Subject Headings and other free-text words in each database. The complete search strategy, including the use of MeSH and free-text words, is provided in Additional file 1.

### Inclusion and exclusion criteria for literature

After excluding duplicates, two authors performed an initial screening of the articles by reading the abstracts, and the remaining articles were assessed for inclusion or exclusion. The reasons for exclusion were recorded. In cases of disagreement on inclusion or exclusion, a third author adjudicated the study, and a final decision was reached after discussion. The inclusion criteria were as follows: (1) Original research, such as prospective studies, retrospective studies, and randomized controlled trials (2) patients who were diagnosed with AVM and had received at least one treatment such as surgery, radiosurgery or embolization; (3) post-treatment follow-up assessments utilizing ASL, with subsequent validation of ASL outcomes against the benchmark provided by DSA; (4) studies that provided comprehensive data, enabling the extraction of a 2 × 2 contingency table for subsequent data analysis. The exclusion criteria were as follows: (1) repeated inclusion of articles; (2) Non original research, such as reviews, editorials, case reports, conference abstracts, books, and letters; (3) studies whose experimental content did not align with the current research; (4) unable to obtain sufficient data to support data analysis.

### Data extraction

The following data were extracted from the included articles: (1) basic study information, including the article title, authors, publication year, and type of study; (2) patient information, including the total number of patients, mean age and range, sex ratio, treatment methods used, follow-up duration, and time interval between ASL detection and DSA testing; (3) study outcomes, including the number of true positives, true negatives, false positives, and false negatives, from which 2 × 2 contingency tables were constructed. To ensure the accuracy of the data extraction, two researchers independently performed the aforementioned tasks and cross-verified the results. Any discrepancies were reassessed by a third researcher to finalize the data. If key data were unavailable, we contacted the authors of the articles via email to obtain the necessary information. If no response occurred within one month, those results were excluded.

### Research quality assessment

The methodology quality of the included studies was evaluated using the 2011 updated Quality Assessment of Diagnostic Accuracy Studies-2 (QUADAS-2) tool [16], with particular emphasis on four domains: patient selection, index test, reference standard, and flow and timing. Each study was independently evaluated by two researchers, who categorized the risk of bias as low, unclear, or high and clinical applicability concerns as low concern, unclear concern, or high concern. In cases of inconsistent evaluations, a third researcher independently reviewed the assessment, and a final evaluation was determined through discussion. All quality assessment results were tabulated using Review Manager 5.3 software to facilitate subsequent analysis and referencing.

### Statistical analysis

After data extraction, a meta-analysis was conducted using a hierarchical (bivariate mixed-effects) model developed by Van Houwelingen [17, 18]. Calculations of pooled sensitivity, specificity, and 95% confidence intervals were performed using Stata18 software, and forest plots including the heterogeneity index I² were generated. An I² less than 50% indicated low heterogeneity, allowing for combined analysis. If the I² exceeded 50%, indicating substantial heterogeneity, a meta-regression analysis on the covariates included in the studies was conducted. Subsequently, summary receiver operating characteristic (SROC) curves, 95% prediction contours, and 95% confidence contours were drawn, and the area under the curve (AUC) was calculated. Furthermore, publication bias was evaluated using Deeks’ funnel plot and the regression test of asymmetry, which is more appropriate for diagnostic accuracy meta-analysis [19, 20]. A p-value greater than 0.05 indicates no significant bias, while a value less than 0.05 suggests the presence of bias. Finally, sensitivity analysis will be performed using manual exclusion of each data item and recalculation.

## Results

### Characteristics of the included studies

**Fig. 1:**
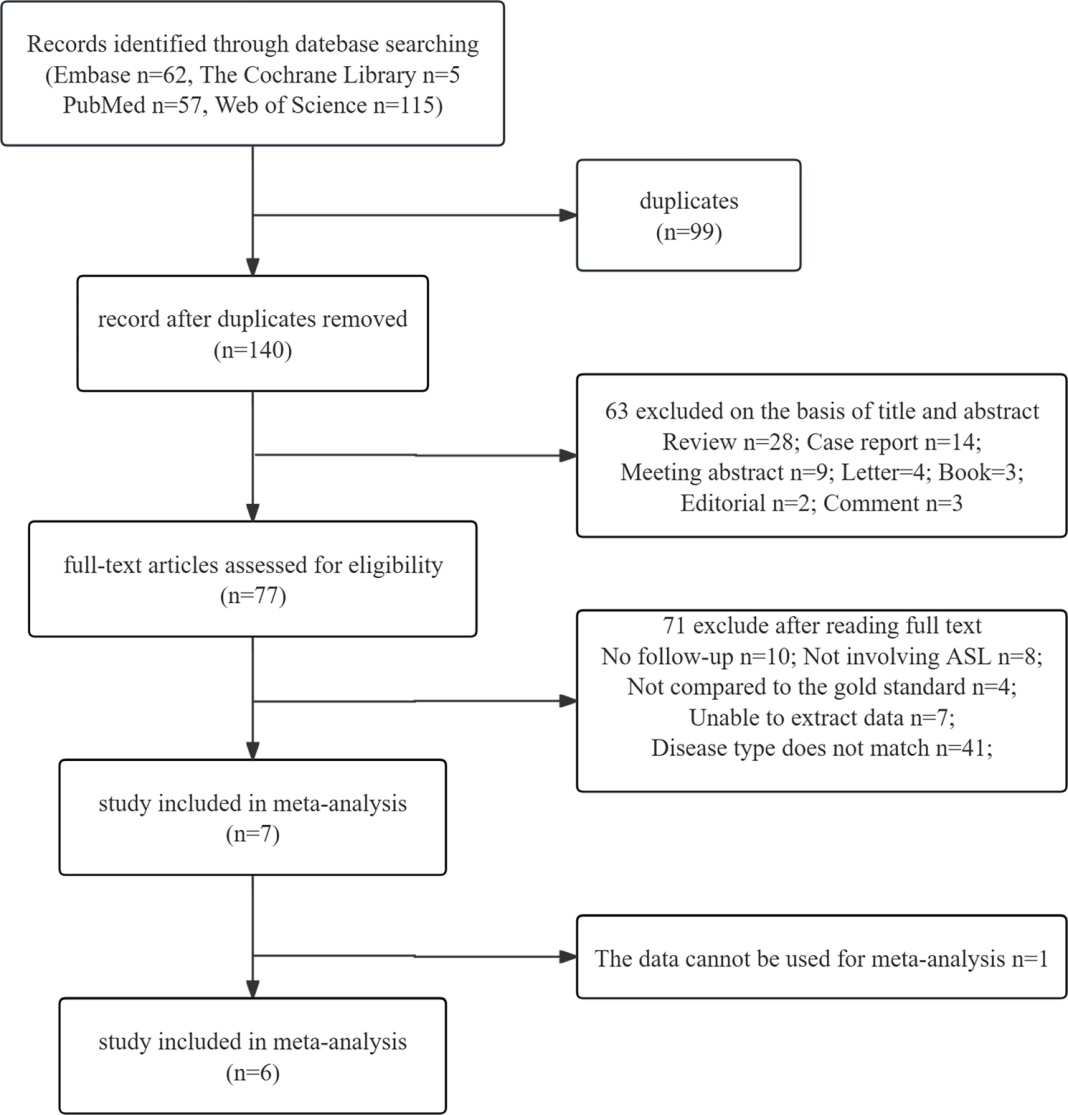
Systematic review and meta-analysis study selection flowchart. This flowchart illustrates the systematic process of identifying, screening, and selecting studies for inclusion in a meta-analysis. The right-hand side boxes detail the specific reasons for exclusion, while the left-hand side boxes indicate the number of articles remaining after each stage of the review process

A total of 138 articles remained after duplicate articles were removed. According to the article titles and abstracts, the full texts of the remaining 45 articles were screened, and 6 articles [21–26] were ultimately included in the meta-analysis. The selection process and reasons for exclusion are illustrated in Fig. 1. This meta-analysis included 132 participants with AVM, ranging in age from 3 to 78 years. The treatments administered to these patients included radiosurgery, surgical operations, embolization therapy, and mixed therapy. The main characteristics of the 6 referenced articles are summarized and tabulated in Tables 1 and 2. Table 3 includes a 2 × 2 contingency table and displays the sensitivity and specificity of each study.

**Table 1:** Basic characteristics of the 6 studies included in this meta-analysis

Author	Year	Study design	Number of patients	Age Mean[range] (year)	Treatment	Include patient information	The size of the patient's AVM Mean[range]	Mean time of follow-up mean[range] (year)
Rojas-V.A. [25]	2021	Prospective	29	37 [18–69]	Radiosurgery	Adult patients undergoing DSA for assessment of obliteration following GKR for AVMs.	7.01 [0.07–50.54 ml]	4.4(3.5–5.3)
Leclerc X. [24]	2020	Retrospective	28	41 [17–65]	Radiosurgery	Patients with a high likelihood of nidus obliteration or small residual shunting.	12 cases:12.5 [5–20] mm 8cases: unmeasured	4.2(2–10)
Wu Chunxue [26]	2021	Retrospective	39	33 [5–64]	Mixed	Patients diagnosed with AVM through DSA, with or without treatment.	SM1:7, SM2:7, SM3:10, SM4:5	
Amponsah K. [21]	2012	Prospective	9	40 [7–78]	Radiosurgery	AVM patients who only receive GKS treatment.	6.58[0.24-40]cm^3^	4.4(2–10)
Jeremy J.H. [22]	2020	Retrospective	15	29 [16–45]	Radiosurgery	Patients undergoing DSA evaluation or SRS treatment for AVM.	SM1:1, SM2:4, SM3:2, SM4:3 SM5:2	2.5(1.35–3.8)
Yuhao Huang [23]	2019	Retrospective	12	10 [3–15]	Mixed	AVM patients with preliminary diagnosis or various intervention treatments.	? [7–30]mm	4.7(2.3–11.6)

**Table 2:** Key parameters in arterial spin labeling (ASL) MRI studies

Author	ASL type		MR field strength	PLD	Gold standard	Interval between test and standard Mean [Range](d)
Rojas-V.A. [25]	PcASL^a^	3T		—	DSA ^c^	< 1d
Leclerc X. [24]	PcASL^b^	3T		2000MS	DSA	10 [1–45]
Wu Chunxue [26]	PcASL	3T		2000MS	DSA	—
Amponsah K. [21]	PASL	—		—	DSA	—
Jeremy J.H. [22]	PcASL	3T/1.5T		2000MS	DSA	39.3[26.5–60]
Yuhao Huang [23]	PcASL	3T		1500MS	DSA	17[2-189]

^a^ PcASL, Pseudocontinuous arterial spin labeling; ^b^ PASL, pulsed arterial spin labeling; ^c^ DAS, digital subtraction angiography

**Table 3:** The diagnostic accuracy of ASL in the 6 included studies

Author	TP^a^	FP^b^	FN^c^	TN^d^	Sensitivity [95%CI^*^]	Specificity [95%CI]
Rojas-V. A. [25]	8	0	1	20	0.89[0.52-1.00]	1.00[0.83-1.00]
Leclerc X. [24]	17	0	3	8	0.85[0.62–0.97]	1.00[0.63-1.00]
Wu Chunxue [26]	27	0	2	10	0.93[0.77–0.99]	1.00[0.69-1.00]
Amponsah K. [21]	4	0	0	5	1.00[0.4-1.00]	1.00[0.48-1.00]
Jeremy J. H. 1 [22]	10	0	0	5	1.00[0.69-1.00]	1.00[0.48-1.00]
Jeremy J. H. 2 [22]	10	0	0	5	1.00[0.69-1.00]	1.00[0.48-1.00]
Jeremy J. H. 3 [22]	10	0	0	5	1.00[0.69-1.00]	1.00[0.48-1.00]
Jeremy J. H. 4 [22]	10	1	0	4	1.00[0.69-1.00]	0.80[0.28–0.99]
Huang Yuhao 1 [23]	8	0	1	3	0.89[0.52-1.00]	1.00[0.29-1.00]
Huang Yuhao 2 [23]	5	0	4	3	0.56[0.21–0.86]	1.00[0.29-1.00]

^a^ TP: true positive; ^b^ FP: false positive; ^c^ FN: false negative; ^d^ TN: true negative; ^*^CI: confidence interval

**Fig. 2:**
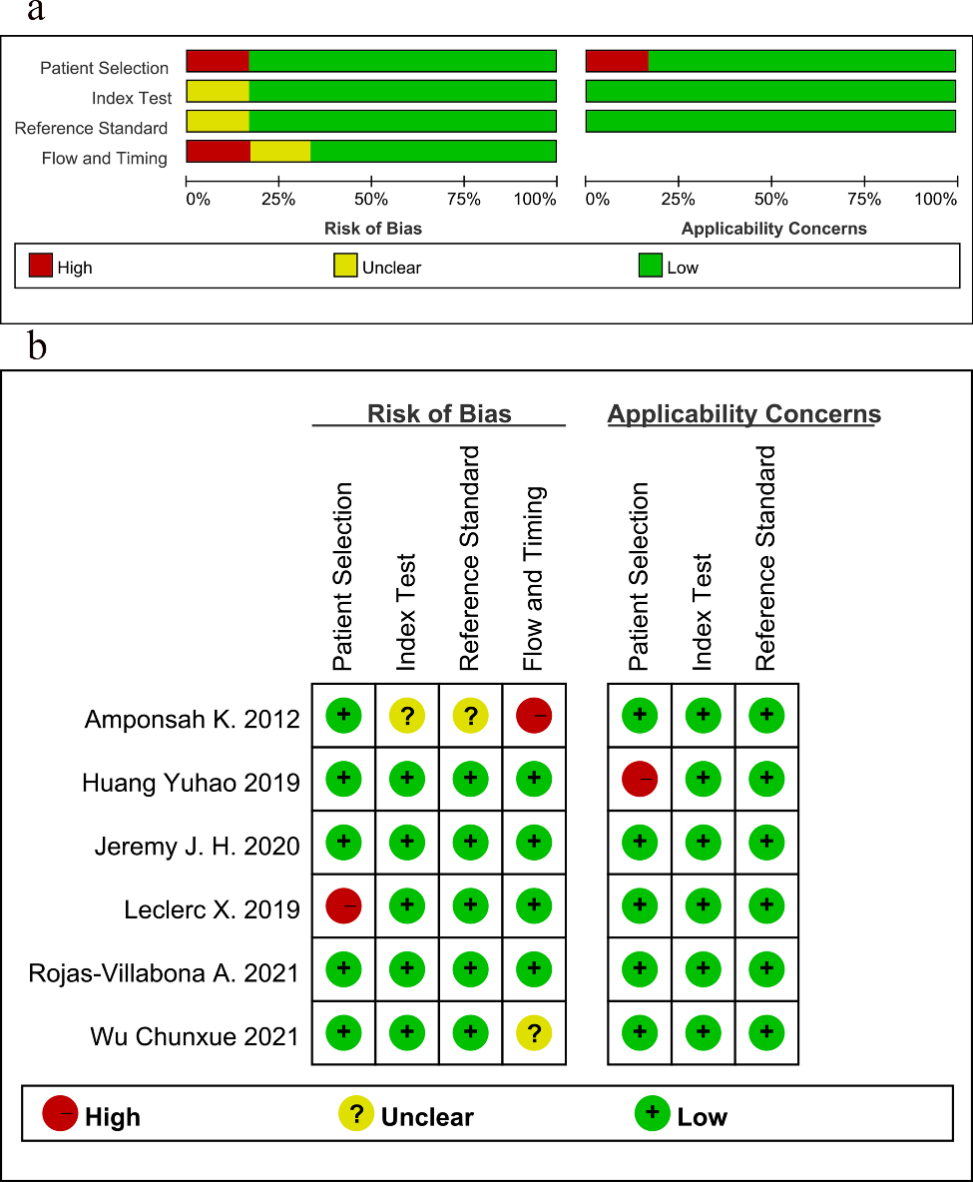
Methodology quality of 7 included studies generated by review manager 5.4. (**a**) Risk of bias and applicability concerns graph. (**b**) Summary of risk bias and applicability concerns. Risk assessments for each study are categorized as “Low Risk” (green), “High Risk” (red), or “Unclear” (yellow). The length of the bars represents the degree of risk across various assessment dimensions

### Quality assessment

We used the QUADAS-2 tool to assess the methodological quality of six articles. The quality of the included studies was visualized and summarized in quality assessment graphs and summary plots (Fig. 2) within Review Manager 5.4. In the domain of patient selection, one study [24] was considered high risk because it exclusively included patients with AVMs smaller than 0.2 mm, which are difficult to diagnose. In the index test domain, one study [21] was assessed as having unclear risk due to the failure to explicitly mention whether blinding was used. Regarding the reference standard, one study [21] had an unclear risk for not specifying whether blinding was used. In the domain of flow and timing, one study was rated as high risk because only some of the included patients underwent the relevant ASL tests. One study [26] was rated as having an unclear risk due to the lack of clear documentation of the follow-up intervals. In the domain of clinical applicability, one study [23] was rated as high risk in patient selection because only it included pediatric patients with AVMs.

### Quantitative synthesis of diagnostic performance

**Fig. 3:**
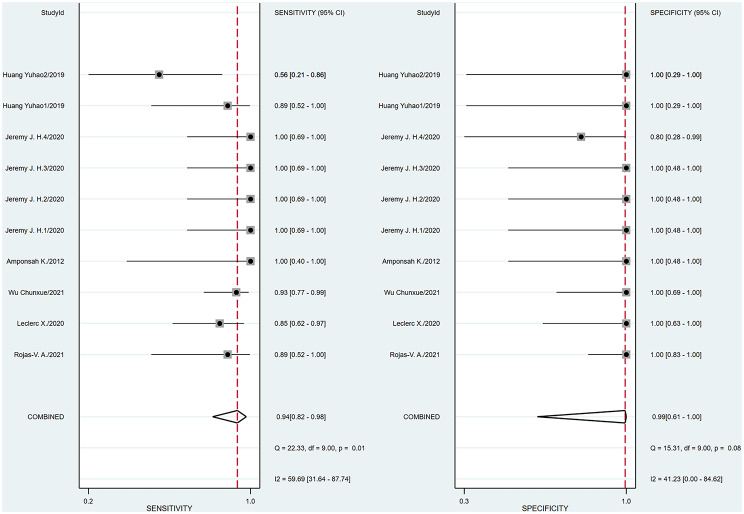
Forest plots of pooled sensitivity and specificity (Include Huang Yuhao2). In the forest plot, each study is represented by a square whose size is proportional to its statistical weight. Each point in the plot represents an individual study’s sensitivity or specificity estimate. The horizontal lines extending from the squares denote the 95% confidence intervals. Red vertical lines represent the pooled sensitivity and specificity of the overall effect estimate. The forest plot demonstrates a high degree of heterogeneity in sensitivity across the studies

**Fig. 4:**
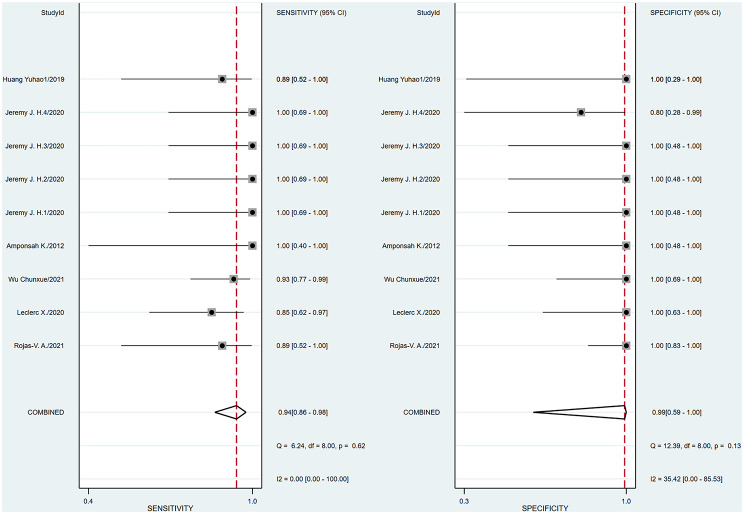
Forest plots of pooled sensitivity and specificity (Exclude Huang Yuhao2). This updated forest plot presents the sensitivity and specificity data for the included studies after excluding outliers that significantly contributed to high heterogeneity. After excluding data that significantly contributed to high heterogeneity, the analysis revealed reduced heterogeneity, with the final pooled sensitivity and specificity estimates being 0.94 and 0.99, respectively

**Fig. 5:**
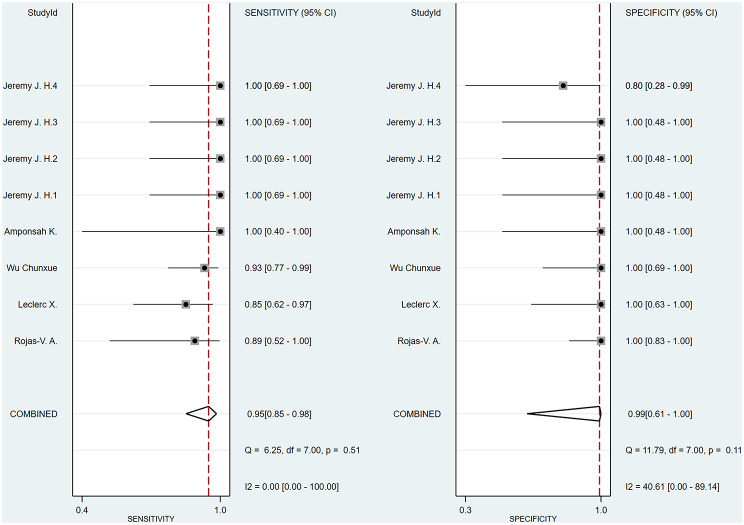
Forest plots of pooled sensitivity and specificity (Exclude Huang Yuhao1-2). Following the exclusion of the study by Huang Yuhao (limited exclusively to pediatric populations), the updated forest plot demonstrated a significant reduction in heterogeneity metrics. Subsequent meta-analysis yielded pooled sensitivity and specificity estimates of 0.95 and 0.99

**Fig. 6:**
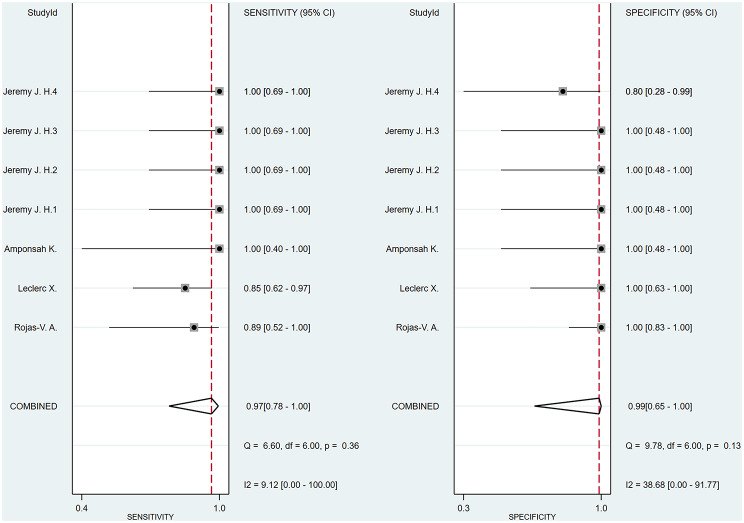
Forest plots of pooled sensitivity and specificity (Exclude Huang Yuhao and Wu chunxue). The reacquired forest plot includes only studies that used a single radiotherapy regimen for intervention, excluding the studies by Huang Yuhao and Wu Chunxue, which involved multiple treatment modalities. The results show a reduction in heterogeneity, with pooled sensitivity and specificity estimates of 0.97 and 0.99, respectively

**Fig. 7:**
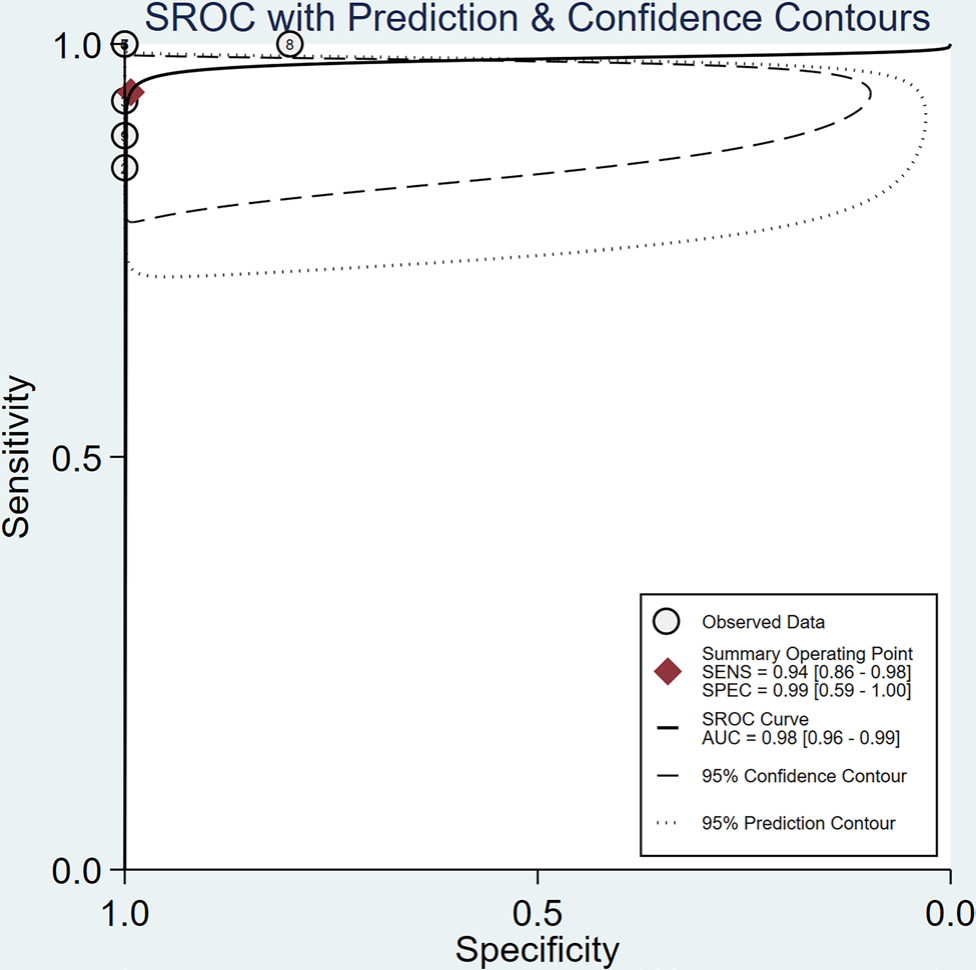
SROC Curve of ASL for Monitoring Treated Cerebral AVM. Each plotted point signifies the sensitivity-specificity pair derived from an individual study, with the red points denoting the aggregated estimates of sensitivity and specificity from the collective analysis of all studies. The area under the curve (AUC), presented as the AUC value, serves as a comprehensive measure of the test’s diagnostic efficacy. The summary operating point indicates sensitivity and specificity of 0.94 and 0.99, respectively. The value of AUC is 0.98 [0.96–0.99]

A bivariate model was employed for the meta-analysis, synthesizing data from six studies. Statistical analysis and data visualization were conducted using Stata 18.0. In two studies, multiple radiologists interpreted ASL images, yielding disparate results that were documented and analyzed individually. Figure 3 presents a forest plot that reveals heterogeneity, pinpointing the specific data contributing to the significant variability. Consequently, the data were excluded, and a subsequent reanalysis was performed, with the updated results shown in Fig. 4. The sensitivity and specificity of the final merger were excellent, with values of 0.94 [0.86–0.98] and 0.99 [0.59-1.00], respectively. The I² values for sensitivity and specificity are 0 [0.00-100] and 35.42 [0.00-85.53], respectively, with p-values of 0.62 and 0.13, respectively. After removing that item, the heterogeneity of the remaining studies is relatively low. We conducted heterogeneity analysis through sequential exclusions. First, removing studies with participants under 18 years (Fig. 5) demonstrated reduced heterogeneity: sensitivity I²=0 [0.00-100] (*p* = 0.51); specificity I²=40.61 [0.00-89.14] (*p* = 0.11). Further exclusion of mixed-treatment studies (Fig. 6) showed improved consistency: sensitivity I²=9.12 [0.00-100] (*p* = 0.36); specificity I²=38.68 [0.00-91.77] (*p* = 0.13). Figure 7 shows the SROC curve with prediction and confidence contours, with observed data points closely surrounding the SROC curve and calculated AUC of 0.98 [0.96–0.99].

### Publication bias and sensitivity analysis

**Fig. 8:**
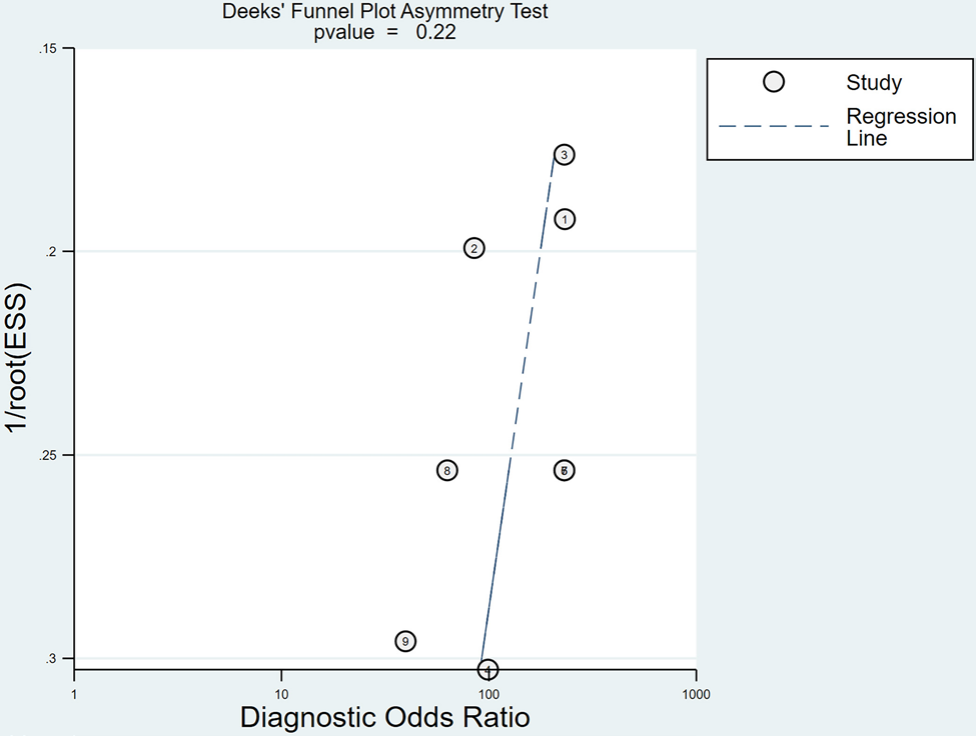
Deeks’ funnel plot to test publication bias. Each data point represents a study whose position is based on its effect size estimate and standard error. The regression line shows the expected location of studies without bias. With a p-value of 0.22, which is above the 0.05 threshold, the analysis does not indicate significant publication bias

Using Stata 18.0, we constructed the Deeks’ funnel plot, which is displayed in Fig. 8. The regression line coefficient resulted in a p-value of 0.22, showing no substantial evidence of publication bias in the study. Manual exclusion of one study followed by reanalysis of the data yielded results similar to those of the original study. Similar results were obtained after repeating the above process, suggesting high stability in the findings of this research.

## Discussion

This meta-analysis showed that the combined sensitivity and specificity of ASL for detecting residual AVMs compared to DSA were 94% and 99%, respectively. This result demonstrates that ASL can effectively identify AVM patients with low rate of missed diagnosis. The SROC curve shows an AUC of 0.98, which further confirms the excellent discriminative ability of ASL. However, considering that the included patients are all AVM patients after treatment, it may lead to a higher specificity. Meanwhile in a similar study [27], which only included AVM patients, the diagnosis rate of AVM by ASL was 0.99. Given the findings, we posit that the determined specificity within our AVM-exclusive study cohort requires additional validation. Notably, the integrated analysis of diagnostic accuracy metrics (sensitivity and specificity), ROC characterization (AUC = 0.98), and heterogeneity patterns of this study provide robust validation of the findings.

Six high-quality studies included patients aged 3–80 years without age restrictions, enhancing sample representativeness while contributing to observed heterogeneity. Following Huang Yuhao’s methodology [23], which exclusively enrolled pediatric AVM cohorts under 18 years of age, we excluded this group from the analysis. This targeted exclusion reduced heterogeneity, yielding a pooled sensitivity of 95% and specificity of 99% in adult populations. This demonstrates enhanced diagnostic accuracy of ASL in adults compared to pediatric patients. While Leclerc X. [24] focused solely on AVM patients with lesions less than 20 mm in diameter. This difference in patient selection criteria contributed to the increased heterogeneity observed in the study. As depicted in Table 3, the sensitivity values for the two groups were comparatively lower than those of other groups. However, the final analysis indicated that the level of heterogeneity was within acceptable limits. This study further confirms the applicability of ASL in different age groups and case situations, emphasizing its significance for clinical practice.

Table 1 summarizes the therapeutic regimens across studies, showing mixed-treatment protocols (surgery or radiosurgery) in the studies by Wu Chunxue [26] and Yuhao Huang [23], and radiosurgery-only approaches in other groups. This methodological divergence emerged as a significant contributor to heterogeneity. Restricting analysis to radiosurgery-exclusive studies significantly attenuated heterogeneity indices, yielding pooled sensitivity of 97% and specificity of 99%. These results suggest that arterial spin labeling (ASL) may provide more accurate diagnostic information following radiosurgery, potentially attributable to reduced confounding from surgical artifact and inflammatory changes. Table 1 also provides a summary of the follow-up durations for each group, with an average of approximately 4 years. The shortest follow-up duration recorded was 1.35 years, and the longest was 11.6 years. The detection of AVMs post-treatment is a process that is both lengthy and crucial. Based on the data presented, we advocate for a minimum follow-up period of at least 4 years. For pediatric patients, considering their rapid growth and developmental changes, we recommend an extended follow-up duration to adequately monitor their condition.

Moreover, six research articles included two arterial spin labeling techniques commonly employed in clinical settings: pulsed arterial spin labeling (PASL) and pseudocontinuous arterial spin labeling (PCASL). While PASL demonstrated perfect sensitivity and specificity (100%) in a small post-treatment cohort (*n* = 9), its broader applicability is constrained by inherent technical limitations. The PASL technique employs short pulses to magnetically label proximal arterial blood, offering distinct advantages including rapid acquisition times and enhanced resistance to magnetic susceptibility artifacts in heterogeneous tissue interfaces [28]. However, its labeling efficiency is limited by arterial transit time (ATT), which may lead to underestimation of cerebral blood flow (CBF) [29]. PCASL has been recommended as the preferred clinical modality due to its implementation of prolonged labeling durations, which achieve superior signal-to-noise ratios (SNR). This approach can theoretically increase the signal-to-noise ratio (SNR) of the ASL sequence by a factor of $\:\sqrt{2}$, and enhance the reliability of quantifying high-flow AVM hemodynamics, particularly at 3T field strength [30].

Furthermore, given the pivotal role of postlabeling delay (PLD) in ASL, future work will aim to optimize the PLD. The appropriate selection of PLD is crucial because it directly influences the accurate measurement of cerebral blood flow or both cerebral blood flow (CBF) and arterial transit time (ATT) [31]. Ideally, the PLD should be slightly longer than the longest ATT observed among subjects. However, due to the decay of the ASL signal with time constant T1 after labeling, an overly conservative selection of PLD can significantly degrade the SNR [32], especially in cases of arterial occlusion or abnormal circulation [12], thus making the choice of PLDs a delicate balancing act. Clinically recommended PLD values are 2000 ms for adults and 1500 ms for children [32]. In these studies, a single delay marking was used, with most studies adhering to the clinically recommended PLD of 2000 ms, while studies involving children with AVMs opted for a PLD of 1500 ms. As medical technology advances, a technique known as multidelay arterial spin labeling (MDASL) has been developed that allows for multiple acquisitions at various PLDs. Utilizing multiple PLDs can help correct for ATT, thereby enhancing the accuracy of CBF measurements and improving the precision of AVM diagnosis [30]. Nevertheless, MDASL faces practical challenges, including practical limitations in imaging time, the SNR, and the complexity of calculation and measurement, which currently limits its routine clinical application. With ongoing developments and optimizations, ASL technologies are becoming increasingly prevalent in clinical practice as innovative diagnostic tools offering high diagnostic accuracy and minimal adverse effects.

However, there are several unavoidable factors in six research articles, including technological limitations, radiation-induced damage, and errors associated with extended time intervals, resulting in biases. For instance, in patients with AVMs, the complete embolization rate is significantly lower due to factors such as the tortuosity of intracranial feeding arteries, the smaller size of the femoral artery access site, and the limited volume of contrast agent [33, 34]. Furthermore, AVMs that are only partially embolized might be misdiagnosed as completely embolized due to significantly reduced blood flow, thereby increasing diagnostic complexity. Besides, damage induced by radiation therapy, such as thrombosis, fibrin exudates, and the dilation and twisting of capillaries and vessels [35]—especially radiation-induced changes that occur within one to two years post-treatment [36–40], and edema or cysts appearing around the lesion after five years or more [41]—may be mistaken for residual brain AVMs, further complicating the diagnosis. Moreover, the occlusion of AVMs is a protracting process [36]; in the studies analyzed, the longest interval between ASL and the gold standard DSA reached 180 days, during which time partially occluded AVMs may gradually become completely occluded, potentially further diminishing the sensitivity and specificity of ASL in diagnosing AVMs.

This study also has several limitations, the most significant being the low prevalence of the target disease, resulting in a limited sample size. Variations in treatment protocols, participant characteristics, and treatment methods among the studies also contributed to variability in the results. Given the limited number of related studies, we were unable to conduct deeper subgroup analyses to more precisely investigate the specific factors influencing the diagnosis of AVM using ASL. Notwithstanding the limitations, this investigation demonstrates distinct methodological merits. First, the study not only assessed the sensitivity and specificity of ASL in diagnosing AVMs, but also systematically evaluated how critical clinical variables— including patient age, lesion size, therapeutic modalities, follow-up duration, and ASL technical parameters—influence diagnostic accuracy. Second, as a comprehensive study, it provides new methods for future research. The findings suggest that ASL has high diagnostic accuracy in adult AVM patients during follow-up after radiosurgery and identify feasible follow-up durations. Finally, this study guides future research focused on analyzing the impact of variables such as age and therapeutic approaches on the diagnostic accuracy of ASL, and on optimizing arterial spin labeling (ASL) applications for diagnosing pediatric AVM patients and small AVMs.

## Conclusion

Although, current research has confirmed that ASL has a high accuracy rate in the diagnosis of AVM, its technology is not yet fully mature. Additional file 2 underscores three critical constraints hindering the widespread implementation of ASL: inherent technical complexities, stringent patient cooperation requirements, and the substantial costs associated with high-field MRI systems. Consequently, ASL cannot completely replace the gold standard DSA at present. However, given that ASL can nearly avoid surgical trauma and the risks associated with contrast agents, decrease radiation exposure and related adverse reactions, ASL has the potential to serve as a valuable adjunctive diagnostic tool in the follow-up care after AVM treatment. In Additional file 2, we also present a comparative analysis of artificial intelligence-based diagnostic approaches. With the continued advancement of ASL technology and AI-driven diagnostic systems, we anticipate that an integrated approach combining ASL, DSA, and AI will emerge as the mainstream diagnostic paradigm. This synergistic methodology has the potential to substantially enhance diagnostic efficiency and accuracy through comprehensive hemodynamic assessment and intelligent pattern recognition.

## Electronic supplementary material

Below is the link to the electronic supplementary material.

Supplementary Material 1

## Data Availability

All data generated or analyzed during this study are included in this published article and its supplementary information files. The complete search strategy, including the use of MESH and free-text words, is provided in the Additional file 1.

## Abbreviations

AVM: Arteriovenous malformation
EVT: Endovascular treatment
DSA: Digital subtraction angiography
ASL: Arterial spin labeling
QUADAS-2: Quality assessment of diagnostic accuracy studies-2
SROC: Summary receiver operating characteristic
AUC: Area under the curve
PASL: Pulsed arterial spin labeling
PcASL: Pseudocontinuous arterial spin labeling
CI: Confidence interval
SNR: Signal-to-noise ratio
CBF: Cerebral blood flow
ATT: Arterial transit times
MDSAL: Multidelay arterial spin labeling
AI: Artificial intelligence
