# Supplementary material for: Meta-analysis of arterial spin labeling MRI to identify residual cerebral arteriovenous malformations after treatment
Source: BMC Med Imaging. 2025 Apr 18;25:127. doi: 10.1186/s12880-025-01668-3 (PMC12007328; doi:10.1186/s12880-025-01668-3)
Supplement: Supplementary file 1 — Supplementary Material 1 [file 12880_2025_1668_MOESM1_ESM.pdf]

Additional file 1 Search strategies

Database	Search strategies	The number of records
PubMed	(("Intracranial Arteriovenous Malformations"[Mesh]) OR (((((((((((((((Arteriovenous Malformation, Intracranial[Title/Abstract]) OR (Arteriovenous Malformations, Intracranial[Title/Abstract])) OR (Intracranial Arteriovenous Malformation[Title/Abstract])) OR (Malformation, Intracranial Arteriovenous[Title/Abstract])) OR (Malformations, Intracranial Arteriovenous[Title/Abstract])) OR (Congenital Intracranial Arteriovenous Malformations[Title/Abstract])) OR (Intracranial Arteriovenous Malformations, Congenital[Title/Abstract])) OR (AVM (Arteriovenous Malformation) Intracranial[Title/Abstract])) OR (Intracranial Arteriovenous Malformation, Ruptured[Title/Abstract])) OR (Ruptured Intracranial Arteriovenous Malformation[Title/Abstract])) OR (Arteriovenous Malformations, Cerebral[Title/Abstract])) OR (Arteriovenous Malformation, Cerebral[Title/Abstract])) OR (Cerebral Arteriovenous Malformation[Title/Abstract])) OR (Malformation, Cerebral Arteriovenous[Title/Abstract])) OR (Malformations, Cerebral Arteriovenous[Title/Abstract])) OR (Cerebral Arteriovenous Malformations[Title/Abstract]))) AND (((((((((((arterial spin labeling) OR (spin labeling)) OR (spin label)) OR (arterial spin label)) OR (asl)) OR (pcasl)) OR (3d pcasl)) OR (3d asl)) OR (4d asl)) OR (4d trance)) OR (pasl)) OR (casl))	57
Web of Science	(((((((((((TS=(arterial spin label)) OR TS=(arterial spin labeling)) OR TS=(spin labeling)) OR TS=(spin label)) OR TS=(asl)) OR TS=(pcasl)) OR TS=(casl)) OR TS=(pasl)) OR TS=(3d pcasl)) OR TS=(3d asl)) OR TS=(4d asl)) AND ((((((((TS=(Intracranial Arteriovenous Malformations)) OR TS=(Intracranial Arteriovenous Malformation)) OR TS=(Congenital Intracranial Arteriovenous Malformations)) OR TS=(Arteriovenous Malformation Intracranial)) OR TS=(AVM Intracranial)) OR TS=(Ruptured Intracranial Arteriovenous Malformation)) OR TS=(Cerebral Arteriovenous Malformation)) OR TS=(Cerebral Arteriovenous Malformations))	115
The Cochrane Library	#1 MeSH descriptor: [Intracranial Arteriovenous Malformations] explode all trees 118<br>#2 (Intracranial Arteriovenous Malformations):ti,ab,kw OR (Malformation, Cerebral Arteriovenous):ti,ab,kw OR	5

	(Malformations, Cerebral Arteriovenous):ti,ab,kw OR (Cerebral Arteriovenous Malformations):ti,ab,kw OR (Arteriovenous Malformations, Cerebral):ti,ab,kw 208<br>#3 (Cerebral Arteriovenous Malformation):ti,ab,kw OR (Cerebral Arteriovenous Malformation):ti,ab,kw OR (AVM (Arteriovenous Malformation) Intracranial):ti,ab,kw OR (Intracranial Arteriovenous Malformations, Congenital):ti,ab,kw OR (Congenital Intracranial Arteriovenous Malformations):ti,ab,kw 116<br>#4 (Arteriovenous Malformation, Intracranial):ti,ab,kw OR (Malformations, Intracranial Arteriovenous):ti,ab,kw OR (Malformation, Intracranial Arteriovenous):ti,ab,kw OR (Arteriovenous Malformations, Intracranial):ti,ab,kw OR (Intracranial Arteriovenous Malformation):ti,ab,kw 171<br>#5 (Intracranial Arteriovenous Malformation, Ruptured):ti,ab,kw OR (Ruptured Intracranial Arteriovenous Malformation):ti,ab,kw 12<br>#6 #1 OR #2 OR #3 OR #4 OR #5 220<br>#7 (arterial spin labeling):ti,ab,kw OR (spin labeling):ti,ab,kw OR (spin label):ti,ab,kw OR (arterial spin label):ti,ab,kw OR (asl):ti,ab,kw 767<br>#8 (pcasl):ti,ab,kw OR (3d pcasl):ti,ab,kw OR (3d asl):ti,ab,kw OR (4d asl):ti,ab,kw OR (4d trance):ti,ab,kw 77<br>#9 (pasl):ti,ab,kw OR (casl):ti,ab,kw 70<br>#10 #7 OR #8 OR #9 794<br>#11 #6 AND #10 5	
Embase	#1 'brain arteriovenous malformation'/exp<br>#2 'arteriovenous aneurysm, brain':ab,ti OR 'arteriovenous cerebral aneurysm':ab,ti OR 'arteriovenous fistula, brain':ab,ti OR 'brain aneurysm, arteriovenous':ab,ti OR 'brain arteriovenous aneurysm':ab,ti OR 'brain arteriovenous fistula':ab,ti OR 'brain arteriovenous shunt':ab,ti OR 'cerebral aneurysm, arteriovenous':ab,ti OR 'cerebral arteriovenous fistula':ab,ti OR 'cerebral arteriovenous malformation':ab,ti OR 'cerebral arteriovenous malformations':ab,ti OR 'intracranial arteriovenous malformations':ab,ti OR 'brain arteriovenous malformation':ab,ti<br>#3 #1 OR #2<br>#4 'arterial spin labeling'/exp<br>#5 'arterial spin label imaging':ab,ti OR 'arterial spin label mri':ab,ti OR 'arterial spin label perfusion imaging':ab,ti OR 'arterial spin label perfusion weighted imaging':ab,ti OR 'arterial spin label technique':ab,ti OR 'arterial spin labeling	62

	imaging':ab,ti OR 'arterial spin labeling magnetic resonance imaging':ab,ti OR 'arterial spin labeling method':ab,ti OR 'arterial spin labeling mri':ab,ti OR 'arterial spin labeling technique':ab,ti OR 'arterial spin labelling method':ab,ti OR 'arterial spin labelling':ab,ti OR 'arterial spin labelling technique':ab,ti OR 'arterial spin tag labeling':ab,ti OR 'arterial spin tagging':ab,ti OR 'asl perfusion mri':ab,ti OR 'asl technique':ab,ti OR 'asl mri':ab,ti OR 'arterial spin labeling':ab,ti<br>#6 #4 OR #5<br>#7 #3 AND #6	

Additional file 2 Technical Comparison

Dimension	ASL (Arterial Spin Labeling)	DSA (Digital Subtraction Angiography)	AI (Artificial Intelligence)
**Diagnostic Capability**	Moderate sensitivity/specificity	Gold standard	Dependent on input data quality
**Patient Safety**	Non-invasive, no radiation, no contrast agent	Invasive, ionizing radiation, risk of contrast agent	Safety depends on underlying imaging modality
**Cost**	Low<br>Costs stem from high-field MRI (3T equipment)	Moderate<br>Costs from catheter consumables, contrast agents, hospitalization	High<br>Costs from high development expenses
**Logistical Challenges**	Requires standardized PLD protocol (lack of consensus); sensitive to motion artifacts	Requires catheter lab and interventional team	Data privacy and cross-institutional sharing barriers; model generalizability limited by training data diversity
**Technical Expertise Required**	MRI physics knowledge (ASL parameter optimization); hemodynamic analysis experience	Qualifications in interventional radiology, catheter manipulation skills	AI programming skills for algorithm development, multidisciplinary collaboration (radiology + data science) for clinical validation
**Scalability**	Moderate<br>Limited 3T equipment availability	Low<br>Uneven distribution of interventional physicians)	High<br>Potential to assist primary care hospitals)
